# Associations between Gene Expression Variations and Ovarian Cancer Risk Alleles Identified from Genome Wide Association Studies

**DOI:** 10.1371/journal.pone.0047962

**Published:** 2012-11-02

**Authors:** Hua Zhao, Jie Shen, Dan Wang, Steven Gregory, Leonardo Medico, Qiang Hu, Li Yan, Kunle Odunsi, Shashikant Lele, Song Liu

**Affiliations:** 1 Department of Cancer Prevention and Controls, Roswell Park Cancer Institute, Buffalo, New York, United States of America; 2 Department of Biostatistics, Roswell Park Cancer Institute, Buffalo, New York, United States of America; 3 Department of Gynecologic Oncology, Roswell Park Cancer Institute, Buffalo, New York, United States of America; University of Iowa, United States of America

## Abstract

Functional genetic variations play important roles in shaping phenotypic differences among individuals through affecting gene expression, and thus, very likely to influence disease susceptibility, such as cancer susceptibility. One critical question in this era of post-genome wide association studies (GWAS) is how to assess the functional significance of the genetic variations identified from GWAS. In the current study, with lymphoblastoid cell lines (LCLs) from 74 non-related women with familial ovarian cancer and 47 unrelated controls matched on gender and race, we explored the associations between seven ovarian cancer risk variants identified from GWAS (*rs3814113* on 9p22.2, *rs2072590* on 2q31, *rs2665390* on 3q25, *rs10088218*, *rs1516982*, *rs10098821* on 8q24.21, and *rs2363956* on 19p13) and whole genome mRNA expression profiles. We observed 95 significant trans-associations at a permutation level of 0.001. Compared to the other risk variants, *rs10088218*, *rs1516982*, and *rs10098821* on 8q24.21 had the greatest number of significant associations (25, 16, and 38, respectively). Two possible cis-associations were observed between *rs10098821* and *c-Myc*, and *rs2072590* and *HS.565379* (Permutated P = 0.0198 and 0.0399, respectively). Pathway enrichment analysis showed that several key biological pathways, such as cell cycle (P = 2.59×10^−06^), etc, were significantly overrepresented. Further characterization of significant associations between mRNAs and risk alleles might facilitate understanding the functions of GWAS discovered risk alleles in the genetic etiology of ovarian cancer.

## Introduction

Recently, genome wide association studies (GWAS) have successfully identified a number of genetic variations which confer risk to human cancer [1]–[3]. However, most of the risk variants identified from GWAS reside in intergenic, intronic, and other non-coding regions of the genome [4]. Therefore, the observed associations have yet to be translated into a full understanding of the genes and genetic elements mediating disease susceptibility. How to study the functional significance of these GWAS hits poses a big challenge in this post-GWAS era. One of the options might be the investigation of the genetics of gene expression. Several landmark studies have unequivocally shown that many transcripts in the human genome are influenced by inherited variation [5]–[9]. Functional genetic variation, which leads to gene expression changes, may play a critical role in determining phenotypic differences among individuals, and thus, is very likely to influence disease susceptibility. As such, studying the associations between genetic variation and gene expression could potentially help prioritize fine-mapping efforts and provide a shortcut to disease biology.

Epithelial carcinoma of the ovary is one of the most common gynecologic malignancies in women [10]. Family history is the strongest risk factor for ovarian cancer. Compared to a 1.6% lifetime risk of developing ovarian cancer in the general population, women with one first-degree relative with ovarian cancer have a 5% risk. Familial clustering with an autosomal dominant pattern of inheritance (hereditary ovarian cancer) results from germ-line mutations in putative tumor suppressor genes (TSGs), such as the *BRCA1/2* and *MLH1/MSH2* genes [11]–[14]. However, known mutations in *BRCA1/2* and mismatch repair (*MMR*) genes can only explain a small part of the familial aggregation of ovarian cancer (5–13%). This suggests that other genetic events may contribute to familial ovarian cancers. Several GWAS have been done in ovarian cancer and several risk variants have been identified, including *rs3814113* on 9p22, *rs2072590* on 2q31, *rs2665390* on 3q25, *rs10088218*, *rs1516982*, *rs10098821* on 8q24, and *rs2363956* on 19p13 [1]–[3]. However, the functional significance of these risk variants is largely unknown. Thus, studying the associations between gene expression and ovarian cancer risk alleles identified from GWAS might help connect risk variants to their putative target genes/transcripts and biological pathways.

To study the associations between gene expression and ovarian cancer risk alleles, we obtained the whole genome mRNA expression profiles in 121 non-redundant lymphoblastoid cell lines (LCLs) derived from 74 non-related familial ovarian cancer patients who are non-carriers of known *BRCA1/2* and *MMR* gene mutations, as well as 47 non-cancer unrelated family controls. We genotyped seven ovarian cancer risk variants discovered from GWAS in these 121 cell lines and studied their associations with gene expression variations. To our knowledge, this is the first genome-wide study to evaluate the associations between mRNA expression variations in LCLs of familial ovarian cancer cases and GWAS discovered ovarian cancer risk alleles [1]–[3].

## Results

Lymphoblastoid cell lines were derived from the blood samples of 74 non-related women with familial ovarian cancer and 47 un-related cancer-free controls recruited for the GRFOCR (see Methods). Gene expression profiles were generated using the Illumina human HT-12 v3 Expression BeadChips. We filtered the processed data to include genes with expression above the background in at least 25% of the samples (n = 121). A total of 10,435 mRNA genes were retained for further analysis.

For each sample, the seven variants identified from three ovarian cancer GWAS were genotyped using the StepOnePlus™ Real Time PCR system and Assays-on-Demand SNP Genotyping products (see Methods). We assessed the potential implications of these GWAS-discovered variants in ovarian cancer, by performing association analysis to analyze the correlations between mRNA expression variations and variant genotypes. Significant associations were identified by evaluating the relationships between variations of mRNA expression levels (with age and case-control status adjusted) and variant genotypes through 10,000 permutations. The number of significant associations at permutation level threshold of 0.05, 0.01 and 0.001 was summarized in **Table 1**. The list of selected top-ranked significant associations (permutated P ≤ 0.001 and r^2^≥0.095) is shown in **Table 2**. One of the most significant associations is observed between *rs10098821* and *IER3* gene (permutated P<0.0001). *IER3*, is a stress-inducible immediate early response gene, whose functions include cell proliferation and apoptosis regulation. It has been found that this gene is pro-apoptotic in the development of ovarian cancer [15]. *rs10098821* explains about 13% of the variation in *IER3*’s expression level as measured by adjusted r^2^.

**Table 1:** Summary of significant association between GWAS discovered variant genotypes and mRNA gene expression phenotypes.

	rs2072590	rs2665390	rs10088218	rs1516982	rs10098821	rs3814113	rs2363956
P<0.05	585	378	821	618	959	274	394
P<0.01	115	52	194	139	251	46	59
P<0.001	6	5	25	16	38	2	3

**Table 2:** List of Top ranked significant associations between mRNA and variants (P<0.001).

mRNAs	SNPs-ID	*P* –value*	r^2^	mRNAs	SNPs-ID	*P* –value*	r^2^
*HS.571028*	rs2072590	0	0.16	*ARL1*	rs1516982	3.00E−04	0.10
*LRRC41*	rs2072590	0	0.16	*HS.340072*	rs1516982	3.00E−04	0.10
*APIP*	rs2665390	0	0.13	*DOCK11*	rs2072590	4.00E−04	0.12
*ARL1*	rs10088218	0	0.13	*CISD2*	rs2665390	4.00E−04	0.10
*ENG*	rs10088218	0	0.11	*KCNMB1*	rs10088218	4.00E−04	0.12
*FLJ21438*	rs10088218	0	0.10	*PIK3C2B*	rs10088218	4.00E−04	0.10
*TMTC4*	rs10088218	0	0.12	*CD226*	rs10098821	4.00E−04	0.10
*GEMIN4*	rs10098821	0	0.13	*HIP1*	rs10098821	4.00E−04	0.10
*IER3*	rs10098821	0	0.13	*MCM7*	rs10098821	4.00E−04	0.10
*VGF*	rs10098821	0	0.13	*RALGPS2*	rs10098821	4.00E−04	0.10
*VGF*	rs10088218	1.00E−04	0.12	*ELMO1*	rs2363956	5.00E−04	0.12
*PLEKHA7*	rs10098821	1.00E−04	0.12	*EHMT1*	rs10098821	5.00E−04	0.10
*ZHX2*	rs10098821	1.00E−04	0.12	*IL32*	rs10098821	5.00E−04	0.10
*CCL4L1*	rs1516982	1.00E−04	0.14	*KCNMB1*	rs10098821	5.00E−04	0.11
*GEMIN4*	rs1516982	1.00E−04	0.10	*RIC8A*	rs10098821	5.00E−04	0.11
*VGF*	rs1516982	1.00E−04	0.13	*RNF44*	rs10098821	5.00E−04	0.10
*PI4K2A*	rs2363956	2.00E−04	0.13	*ODF1*	rs1516982	5.00E−04	0.10
*HS.572064*	rs2072590	2.00E−04	0.13	*TRPC1*	rs2363956	6.00E−04	0.12
*CCL4L1*	rs10088218	2.00E−04	0.11	*MFGE8*	rs10098821	6.00E−04	0.10
*BSDC1*	rs10098821	2.00E−04	0.11	*APOBEC3H*	rs2072590	7.00E−04	0.10
*GART*	rs10098821	2.00E−04	0.11	*EDEM1*	rs2072590	7.00E−04	0.11
*HS.340072*	rs10098821	2.00E−04	0.12	*HS.579631*	rs2665390	7.00E−04	0.10
*MAPKAP1*	rs10098821	2.00E−04	0.11	*NASP*	rs10098821	7.00E−04	0.10
*RGL1*	rs1516982	2.00E−04	0.10	*MT1G*	rs3814113	7.00E−04	0.15
*OPTN*	rs10088218	3.00E−04	0.10	*DAP3*	rs2665390	8.00E−04	0.10
*ZHX2*	rs10088218	3.00E−04	0.10	*FANCE*	rs1516982	8.00E−04	0.10
*CAV1*	rs10098821	3.00E−04	0.11	*GSTP1*	rs1516982	8.00E−04	0.13
*RGL1*	rs10098821	3.00E−04	0.11	*HS.340072*	rs10088218	9.00E−04	0.10
*STC2*	rs10098821	3.00E−04	0.10	*KRT17*	rs10098821	9.00E−04	0.10
*TBXAS1*	rs10098821	3.00E−04	0.10	*ATL2*	rs3814113	9.00E−04	0.11

* Permutated P value.

Interestingly, the three variants from the 8q24 locus, namely *rs10098821*, *rs10088218* and *rs1516982*, had the largest significant associations among all seven variants. At the 0.05 permutation threshold, the number of significant associations with these three variants was 959, 821 and 618. The number was 251, 194 and 139 at the more stringent permutation threshold of 0.01, and 38, 25 and 16 at the threshold of 0.001. These three variants share a number of significant mRNA gene expression associations. At the 0.05 permutation threshold, three hundred and twelve mRNAs, which account for 33% of the mRNA correlated with *rs10098821*, 38% of mRNA correlated with *rs10088218*, and 50% of mRNA correlated with *rs1516982*, are correlated with all three variants (**Figure S1**). For example, levels of *FANCE* (Fanconi anemia, complementation group E) expression is significantly associated with *rs1516982* (permutated P = 8.0×10^−4^, adjusted r^2^ = 10.3%), *rs10098821* (permutated P = 0.0037, adjusted r^2^ = 7.0%) and *rs10088218* (permutated P = 0.0312, adjusted r^2^ = 3.4%), but none of the other four SNPs (**Figure S2**).

**Figure 1:**
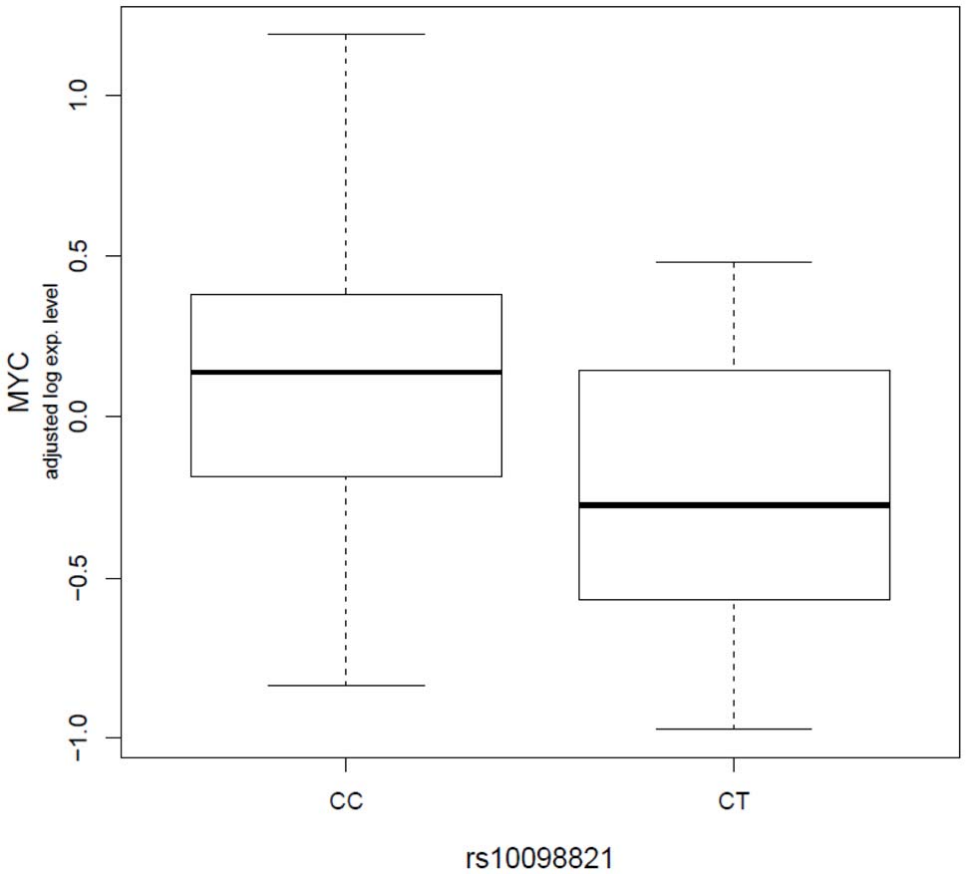
Significant *cis*-associations between *rs10098821* genotype and *c-Myc* expression phenotypes. The boxplot shows the relationship between log_2_ residuals of *c-Myc* expression levels (adjusted for age and case-control status) and genotype of the *rs10098821*. *rs10098821* explained approximately 4.0% of the variation in *c-Myc* expression as measured by adjusted r^2^.

We observed two possible *cis*-associations in which the variant genomic location is within 1 Mb around the probe targeting gene. One *cis*-association is between *rs10098821* and *c-Myc* gene, which is 806 kb away from the variant (permutated P = 0.0198, **Figure 1**), and the other is between *rs2072590* and *HS.565379*, which is 697 kb away from the variant (permutated P = 0.0399, not shown). *rs10098821* explained approximately 4.0% of the variation in *c-Myc* expression as measured by adjusted r^2^. Individuals with T variant alleles have statistically significantly lower expression of *c-Myc* compared to ones without T variant alleles. *rs2072590* explained about 4.4% of the variation in *HS.565379* expression. *HS.565379* has been found to show tissue-specific expression in uterus and uterine tumor based on EST-based gene expression profiling [16].

**Figure 2:**
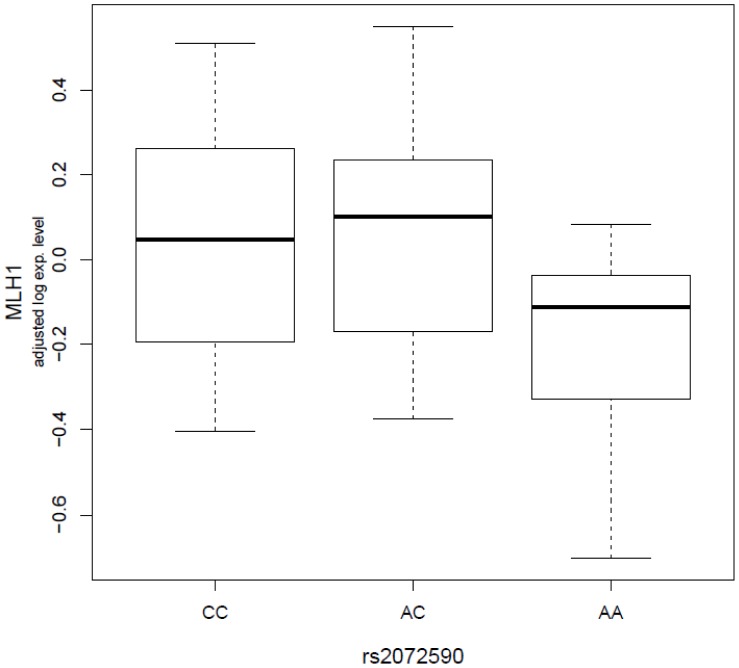
Significant associations between *rs2072590* genotype and *MLH1* expression phenotypes (permutated P = 0.0049, adjusted r^2^ = 8.3%). The boxplot shows the relationship between log_2_ residuals of *MLH1* expression levels (adjusted for age and case-control status) and genotype of the *rs2072590*.

Then, we investigated whether there are any significant associations between these seven variants and known ovarian cancer risk genes, including *BRCA1/2*, *MMR* genes, *p53*, *etc.* We didn’t observe any significant association between these variants and the *BRCA1/2* genes. However, we found several significant associations between the variants and the *MMR* genes and the *p53* gene (**Figure S3**). For example, we found the expression level of the *MLH1* gene is significantly associated with *rs2072590,* a variant on the 2q31 loci (permutated P = 0.0049, **Figure 2**). *rs2072590* explained about 8.3% of the variation in *MLHL1*’s expression level. The expression of the *p53* gene is significantly associated with *rs2665390* (permutated P = 0.018, adjusted r^2^ = 0.036), *rs1516982* (permutated P = 0.028, adjusted r^2^ = 0.035), and *rs10088218* (permutated P = 0.049, adjusted r^2^ = 0.025). Additionally, the expression of the *MSH5* gene is significantly associated with *rs2363956* (permutated P = 0.0056, adjusted r^2^ = 0.075).

Finally, to biologically characterize those mRNA genes significantly associated with GWAS discovered ovarian cancer risk alleles investigated here, we performed Gene Ontology (GO) enrichment analysis using the NCBI DAVID tool [17] As shown in **Table 3**, the list of significantly enriched GO biological processes include “cell cycle” (P = 2.59×10^−6^), “regulation of apoptosis” (P = 4.37×10^−5^), and “programmed cell death” (P = 6.93×10^−5^). At the molecular function level, the significantly enriched GO terms include “nucleotide binding” (P = 7.43×10^−9^), “ATP binding” (P = 3.94×10^−7^), “transcription factor binding” (P = 1.19×10^−5^) and “DNA helicase activity” (P = 3.41×10^−4^).

**Table 3:** Enriched GO Biological Processes for the genes with significant associations with GWAS discovered variants.

GO Term	Count	Size	P-Value	FDR
GO:0006396∼RNA processing	120	547	1.50×10^−8^	6.37×10^−5^
GO:0043933∼macromolecular complex subunit organization	144	710	9.22×10^−8^	1.96×10^−4^
GO:0065003∼macromolecular complex assembly	130	665	3.19×10^−6^	0.003382
GO:0007049∼cell cycle	148	776	2.59×10^−6^	0.003662
GO:0016071∼mRNA metabolic process	81	370	4.50×10^−6^	0.003819
GO:0006259∼DNA metabolic process	102	506	1.11×10^−5^	0.007811
GO:0022613∼ribonucleoprotein complex biogenesis	46	180	1.40×10^−5^	0.008466
GO:0006397∼mRNA processing	70	321	2.49×10^−5^	0.013134
GO:0046907∼intracellular transport	124	657	3.07×10^−5^	0.014409
GO:0043067∼regulation of programmed cell death	147	812	4.75×10^−5^	0.018195
GO:0042981∼regulation of apoptosis	146	804	4.37×10^−5^	0.018393
GO:0010941∼regulation of cell death	147	815	5.80×10^−5^	0.020328
GO:0012501∼programmed cell death	115	611	6.93×10^−5^	0.022407
GO:0006915∼apoptosis	113	602	8.96×10^−5^	0.023497
GO:0008219∼cell death	131	719	9.58×10^−5^	0.023645
GO:0006986∼response to unfolded protein	23	71	7.96×10^−5^	0.023869
GO:0016265∼death	132	724	8.87×10^−5^	0.024805
GO:0034470∼ncRNA processing	44	187	1.73×10^−4^	0.039963
GO:0070271∼protein complex biogenesis	96	505	2.05×10^−4^	0.044756
GO:0006461∼protein complex assembly	96	505	2.05×10^−4^	0.044756
GO:0034660∼ncRNA metabolic process	51	230	2.34×10^−4^	0.048516
GO:0051789∼response to protein stimulus	29	107	2.48×10^−4^	0.048976

## Discussion

The genetic etiology of familial ovarian cancer is still a mystery. Known mutations in *BRCA1/2* and *MMR* genes can only explain a small part of the familial aggregation of ovarian cancer. The results from recent GWAS studies have identified several common genetic variants conferring risk for ovarian cancer [1]–[3]. However, most of these variants are not in protein-encoding regions, so the functional significance of these variants is largely unknown. The current study presents an attempt to dissect the genetic susceptibility of familial ovarian cancer, as well as elucidate the potential functional significance of the identified risk variants from GWAS. Specifically, we investigated the associations between seven significant variants identified from ovarian cancer GWAS and global mRNA expression.

As expected, we have observed a larger number of distant (*trans*-) than local (*cis*-) associations. Among the two identified *cis*-associations, the association between *rs10098821* at 8q24 and *c-Myc* is particularly interesting. Common variants at 8q24 have previously been shown to confer susceptibility to multiple cancer phenotypes, including prostate, colorectal, breast and bladder cancers [18]–[23], and previous functional studies have suggested that common variants in this region may be associated with transcriptional regulation of *c-Myc* [24]–[25]. Most risk associations at 8q24 are located 5′ of *c-Myc*, but the three most significant SNPs for ovarian cancer lie in an apparent gene desert which is >700 kb 3′ of *c-Myc*, suggesting either that *c-Myc* might not be the target susceptibility gene for ovarian cancer or that variants in this region are also capable of distant regulation of *c-Myc*. In a previous study [2], Goode et al compared *c-Myc* expression in 48 normal ovarian epithelial cell lines between individuals without *rs10098821* variant alleles and ones with at least one *rs10098821*variant alleles. Using *GAPDH* as the reference mRNA, they found that the ones without *rs10098821* variant alleles had higher *c-Myc* expression than ones with at least one *rs10098821* variant alleles (Median of relative expression: 0.97 vs 0.62). However, the difference didn’t reach statistical significance (P = 0.43). Similar to their findings, we have observed that individuals without *rs10098821* variant alleles had significantly higher levels of *c-Myc* expression compared to ones with at least one *rs10098821* variant alleles (permutated P = 0.0198). As we have indicated above, *rs10098821* is 3′ of *MYC* and lies about 0.8 Mb away. How this SNP might affect *MYC* expression is still unclear.

Using these identified significant associations in the pathway analysis, we have found that the genes significantly associated with GWAS discovered ovarian cancer risk alleles are enriched in several key biological pathways, such as cell cycle, cellular response to stress/damage, energy metabolism, transcriptional factor binding, *etc*. Interestingly, most known familial ovarian cancer genes (*i.e.*, *BRCA1/2* and *MMR*) are key players in these key pathways. For example, it has been demonstrated that *BRCA1* is the key regulator in sensing DNA stress/damage and subsequently promoting cell cycle arrest [26]. Although our association analysis cannot pinpoint the exact functions of these GWAS discovered variants, it provides a list of potential biological pathways for which one could focus on in future analysis.

There are several limitations to this study. First, many mRNAs are expressed in a tissue-restricted manner. The results from LCLs in this study are likely to represent a small subset of mRNA expression variations. Also, our ability to study the genetics of mRNA expression is limited by the fact that we only investigated seven variants in the analysis, although these seven variants have been associated with ovarian cancer risk in recent GWASs. Second, the effects on transcript abundance may be subtle and therefore below the sensitivity threshold of the microarray platform, and the sample size in our study is relatively small. Third, there is a concern about what the results actually mean when measuring expression in non-tumor tissue at a single point in time. The ultimate goal of our study is to identify the inherited genetic determinants of mRNA expression in normal tissues rather than somatic alterations of mRNA gene expression in tumor tissues. Studies have been shown that at least part of the mRNA gene expression is genetically determined. Therefore, even at a single time-point in non-tumor tissue, what we have observed from this study still provides useful information about how mRNA expression is genetically regulated. Forth, certain effects may only be revealed in certain contexts, such as perturbation of a particular pathway, and may occur through changes in gene transcripts mediated by alterations in microRNAs or non-coding RNAs rather than through direct effects on genes. In these cases, alternative assays will be required to implicate these genes. Finally, the significant associations are not further functionally characterized since all of the top associations are trans-associations. So far, there is still lack of established experimental methods to assess trans-regulation between SNPs and gene expression.

To the best of our knowledge, this study provides the first assessment of the expression level variation of mature human mRNAs in LCLs from familial ovarian cancer patients and healthy unrelated controls. Further studies are needed to identify the genetic causes and biological consequences related to the identified significant associations. Significant associations identified in this study may potentially facilitate better understanding of the genetic etiology of familial ovarian cancer.

## Materials and Methods

### Study Population

This study has been approved by the Institutional Research Board (IRB) of Roswell Park Cancer Institute. Written informed consents have been obtained from all study subjects. Data and samples from women with ovarian cancer and their relatives who were cancer-free were obtained from the Gilda Radner Familial Ovarian Cancer Registry (GRFOCR). Seventy-four non-related women with familial ovarian cancer were included in this study as the cases. They were identified from families with inherited ovarian cancer in which at least two first or second degree relatives had epithelial ovarian cancer diagnosed at any age. All of the women were non-carriers of *BRCA1/2* or *MLH1/MSH2* mutations. Over time, different methods have been used to determine the mutation status of *BRCA1/2* in GRFOCR samples. For samples collected before 2002, mutation status was determined by screening all exons and intron/exon splice junctions of *BRCA 1/2* by a combination of SSCP and HD analysis. Additionally, exon 11 of *BRCA1* was assayed by the protein truncation test for stop codon generating mutations. If alterations were found, the altered fragment was sequenced. Since 2002, sequencing of exons and splice junctions was used. In the last 5 years, all samples (old and new) not showing a mutation were assayed for *BRCA1* large-scale rearrangements. The cancer-free controls of GRFOCR were family relatives of the cases, including mothers, sisters, nieces, *etc.* However, in this study, we chose to use unrelated controls. Unrelated controls are women who are not relatives of any cases used in this study. Forty-seven unrelated controls were included. The cases and controls were matched on gender and race. All of the cases and controls were white women. The median age at cancer diagnosis for the 74 cases was 47 (ranging from 21 to 85), while the median age for the 47 controls at enrollment in GRFOCR was 58 (ranging from 26 to 89). All study subjects donated blood samples when they were enrolled in the GRFOCR. LCLs were established by EBV transformation using the isolated lymphocytes from the blood samples. The study was approved by the institutional IRB board.

### Lymphoblastoid Cell Lines (LCLs) Culture and RNA Extraction

LCLs were maintained in RPMI 1640 (GIBCO BRL) media supplemented with 15% fetal calf serum and antibiotics at 37°C, 5% CO_2_ atmospheric condition and 95% humidity. Total cellular RNAs were isolated from LCLs using TRIzol reagent according to the protocols provided by the manufacturer (Invitrogen Corp., Carlsbad, CA, USA). Purified RNAs were further processed to remove any contaminating DNA (DNA-free kit, Ambion, Inc., Austin, TX, USA). The quality and quantity of the RNA was evaluated by 260/280 ratio using NanoDrop spectrophotometry (NanoDrop ND-1000 Technologies Inc.) and Agilent 2100 Bioanalyzer (Agilent Technologies).

### Genotyping Analysis for Ovarian Cancer Risk Alleles

Seven SNPs, which are identified from 3 ovarian cancer GWAS, were included in the genotyping analysis. They are *rs3814113* on 9p22.2, *rs2072590* on 2q31, *rs2665390* on 3q25 in the intron of TCDD-inducible poly(ADP-ribose) polymerase (*TIPARP*) gene, *rs2363956* on 19p13 in the ankyrin repeat and LEM domain containing 1 (*ANKLE1*) gene, *and rs10088218*, *rs1516982*, and *rs10098821* on 8q24.21. *rs2363956* is a nonsynomous SNP which leads to a Leu to Trp amino acid change. Genotyping analysis was carried out using StepOnePlus™ Real Time PCR system and Assays-on-Demand SNP Genotyping products for fluorogenic polymerase chain reaction allelic discrimination (Applied Biosystems). Each PCR reaction plate included negative controls, positive controls, and unknown samples. The minor allele frequencies for each SNP in the cases and unrelated controls were 0.346/0.298 (P = 0.65) for *rs3814113*, 0.3/0.368 (P = 0.84) for *rs2072590*, 0.081/0.060 (P = 0.60) for *rs2665390*, 0.149/0.107 (P = 0.13) for *rs10088218*, 0.167/0.119 (P = 0.06) for *rs1516982*, 0.127/0.071 (P = 0.07) for *rs10098821*, and 0.432/0.488 (P = 0.20) for *rs2363956*. The genotyping data have been deposited in NCBI’s Gene Expression Omnibus (GEO) with accession number GSE37582.

### Gene Expression Microarray

Two hundred nanograms of total RNA from each sample were labeled and hybridized on Illumina human HT-12 v3 Expression BeadChips according to the manufacturer’s recommendations (Illumina Whole-Genome Gene Expression Guide). The expression profiles have been deposited in NCBI’s Gene Expression Omnibus (GEO) with accession number GSE37582.

### Statistical Analysis

The raw intensity of the Illumina human HT-12 v3expression array was scanned and extracted using BeadScan, with the data corrected by background subtraction in the GenomeStudio module. The lumi package in the R-based Bioconductor Package was used to normalize the log_2_ transformed intensity data by using the Quantile normalization algorithm. For data quality control, we excluded the probes with detection P value>0.05 (the P values were generated in BeadStudio software) in at least 25% (n = 121) of the samples. A total of 10,435 mRNA genes passed the quality control step and were used for downstream analysis. The association of SNP genotype with residuals of expression level adjusted for age and case-control status was calculated using linear regression model as described before (27). Ten thousand permutations of the expression phenotypes relative to SNP genotypes were performed (28–29). To derive P-values adjusted for multiple testing, we determined the percentage of times out of 10,000 permutations that the observed P-value was exceeded in the permuted data analysis.

## Supporting Information

Figure S1 **Venn diagram showing the overlaps of mRNA genes significantly (permutated P<0.05) associated with the three GWAS discovered SNPs from 8q24 locus.**
(TIF)

Figure S2 **Significant associations between the genotypes of the three variants on 8q24 locus and** ***FANCE*** **expression phenotypes.** The boxplot shows the relationship between log_2_ residuals of *FANCE* expression levels (adjusted for age and case-control status) and genotype of the *rs1516982* (top, permutated P = 8.0×10^−4^, adjusted r^2^ = 10.3%), *rs10098821* (middle, permutated P = 0.0037, adjusted r^2^ = 7.0%) and *rs10088218* (bottom, permutated P = 0.0312, adjusted r^2^ = 3.4%).
(TIF)

Figure S3 **Significant associations between the variants genotypes and** ***p53*** **expression phenotypes.** The boxplot shows the relationship between log_2_ residuals of *p53* expression levels (adjusted for age and case-control status) and genotype of the *rs2665390* (top, permutated P = 0.0181, adjusted r^2^ = 3.6%), *rs1516982* (middle, permutated P = 0.0279, adjusted r^2^ = 3.5%) and *rs10088218* (bottom, permutated P = 0.0494, adjusted r^2^ = 2.5%).
(TIF)

## References

[1] Bolton KL, Tyrer J, Song H, Ramus SJ, Notaridou M, et al (2010) Common variants at 19p13 are associated with susceptibility to ovarian cancer. Nat Genet 42: 880–884. 20852633 10.1038/ng.666 PMC3125495

[2] Goode EL, Chenevix-Trench G, Song H, Ramus SJ, Notaridou M, et al (2010) A genome-wide association study identifies susceptibility loci for ovarian cancer at 2q31 and 8q24. Nat Genet 42: 874–879. 20852632 10.1038/ng.668 PMC3020231

[3] Song H, Ramus SJ, Tyrer J, Bolton KL, Gentry-Maharaj A, et al (2009) A genome-wide association study identifies a new ovarian cancer susceptibility locus on 9p22.2. Nat Genet 41: 996–1000. 19648919 10.1038/ng.424 PMC2844110

[4] Freedman ML, Monteiro AN, Gayther SA, Coetzee GA, Risch A, et al (2011) Principles for the post-GWAS functional characterization of cancer risk loci. Nat Genet 43: 513–518. 21614091 10.1038/ng.840 PMC3325768

[5] Monks SA, Leonardson A, Zhu H, Cundiff P, Pietrusiak P, et al (2004) Genetic inheritance of gene expression in human cell lines. Am J Hum Genet 75: 1094–1105. 15514893 10.1086/426461 PMC1182144

[6] Morley M, Molony CM, Weber TM, Devlin JL, Ewens KG, et al (2004) Genetic analysis of genome-wide variation in human gene expression. Nature 430: 743–747. 15269782 10.1038/nature02797 PMC2966974

[7] Stranger BE, Forrest MS, Clark AG, Minichiello MJ, Deutsch S, et al. (2005) Genome-wide associations of gene expression variation in humans. PLoS Genet 1, e78. 10.1371/journal.pgen.0010078 PMC1315281 16362079

[8] Schadt EE, Monks SA, Drake TA, Lusis AJ, Che N, et al (2003) Genetics of gene expression surveyed in maize, mouse and man. Nature 422: 297–302. 12646919 10.1038/nature01434

[9] Johnson JM, Castle J, Garrett-Engele P, Kan Z, Loerch PM, et al (2003) Genome-wide survey of human alternative pre-mRNA splicing with exon junction microarrays. Science 302: 2141–2144. 14684825 10.1126/science.1090100

[10] Yancik R (1993) Ovarian cancer. Age contrasts in incidence, histology, disease stage at diagnosis, and mortality. Cancer 71: 517–523. 8420671 10.1002/cncr.2820710205

[11] Narod SA, Ford D, Devilee P, Barkardottir RB, Lynch HT, et al (1995) An evaluation of genetic heterogeneity in 145 breast-ovarian cancer families. Am J Hum Genet 156: 254–264. PMC1801289 7825586

[12] Ford D, Easton DF, Stratton M, Narod S, Goldgar D, et al (1998) Genetic heterogeneity and penetrance analysis of the BRCA1 and BRCA2 genes in breast cancer families. Am J Hum Genet 62: 676–689. 9497246 10.1086/301749 PMC1376944

[13] Easton DF, Bishop DT, Ford D, Crockfor GF (1993) Genetic linkage analysis in familial breast and ovarian cancer: results from 214 families. Am J Hum Genet 52: 678–701. 8460634 PMC1682082

[14] Lynch HT, Albano WA, Lynch JF, Lynch PM, Campbell A (1982) Surveillance and management of patients at high genetic risk for ovarian carcinoma. Obstet Gynecol 59: 589–596. 7070730

[15] Han L, Geng L, Liu X, Shi H, He W, et al (2011) Clinical significance of IEX-1 expression in ovarian carcinoma. Ultrastruct Pathol 35: 260–266. 22085302 10.3109/01913123.2011.608916

[16] Wheeler DL, Church DM, Federhen S, Lash AE, Madden TL, et al (2003) Database Resources of the National Center for Biotechnology. Nucl Acids Res 31: 28–33. 12519941 10.1093/nar/gkg033 PMC165480

[17] Huang DW, Sherman BT, Lempicki RA (2009) Systematic and integrative analysis of large gene lists using DAVID Bioinformatics Resources. Nature Protoc 4: 44–57. 19131956 10.1038/nprot.2008.211

[18] Yeager M, Chatterjee N, Ciampa J, Jacobs KB, Gonzalez-Bosquet J, et al (2009) Identification of a new prostate cancer susceptibility locus on chromosome 8q24. Nat Genet 41: 1055–1057. 19767755 10.1038/ng.444 PMC3430510

[19] Gudmundsson J, Sulem P, Gudbjartsson DF, Blondal T, Gylfason A, et al (2009) Genome-wide association and replication studies identify four variants associated with prostate cancer susceptibility. Nat Genet 41: 1122–1126. 19767754 10.1038/ng.448 PMC3562712

[20] Al Olama AA, Kote-Jarai Z, Giles GG, Guy M, Morrison J, et al (2009) Multiple loci on 8q24 associated with prostate cancer susceptibility. Nat Genet 41: 1058–1060. 19767752 10.1038/ng.452

[21] Tenesa A, Farrington SM, Prendergast JG, Porteous ME, Walker M, et al (2008) Genome-wide association scan identifies a colorectal cancer susceptibility locus on 11q23 and replicates risk loci at 8q24 and 18q21. Nat Genet 40: 631–637. 18372901 10.1038/ng.133 PMC2778004

[22] Ghoussaini M, Song H, Koessler T, Al Olama AA, Kote-Jarai Z, et al (2008) Multiple loci with different cancer specificities within the 8q24 gene desert. J Natl Cancer Inst 100: 962–966. 18577746 10.1093/jnci/djn190 PMC2902819

[23] Kiemeney LA, Thorlacius S, Sulem P, Geller F, Aben KK, et al (2008) Sequence variant on 8q24 confers susceptibility to urinary bladder cancer. Nat Genet 40: 1307–1312. 18794855 10.1038/ng.229 PMC4539560

[24] Jia L, Landan G, Pomerantz M, Jaschek R, Herman P, et al (2009) Functional enhancers at the gene-poor 8q24 cancer-linked locus. PLoS Genet 5: e1000597. 19680443 10.1371/journal.pgen.1000597 PMC2717370

[25] Pomerantz MM, Ahmadiyeh N, Jia L, Herman P, Verzi MP, et al (2009) The 8q24 cancer risk variant rs6983267 shows long-range interaction with MYC in colorectal cancer. Nat Genet 41: 882–884. 19561607 10.1038/ng.403 PMC2763485

[26] Wu J, Lu LY, Yu X (2010) The role of BRCA1 in DNA damage response. Protein Cell 1: 117–123. 21203981 10.1007/s13238-010-0010-5 PMC3078634

[27] Stranger BE, Forrest MS, Dunning M, Ingle CE, Beazley C, et al (2007) Relative impact of nucleotide and copy number variation on gene expression phenotypes. Science 315: 848–853. 17289997 10.1126/science.1136678 PMC2665772

[28] Stranger BE, Nica AC, Forrest MS, Dimas A, Bird CP, et al (2007) Population genomics of human gene expression. Nat Genet 39: 1217–1224. 17873874 10.1038/ng2142 PMC2683249

[29] Montgomery SB, Sammeth M, Gutierrez-Arcelus M, Lach RP, Ingle C, et al (2010) Transcriptome genetics using second generation sequencing in a Caucasian population. Nature 464: 773–777. 20220756 10.1038/nature08903 PMC3836232

