# Correction: Associations between Gene Expression Variations and Ovarian Cancer Risk Alleles Identified from Genome Wide Association Studies

**DOI:** 10.1371/annotation/febad39e-58f0-4562-a687-1fee2a7d1eff

**Published:** 2013-05-10

**Authors:** Hua Zhao, Jie Shen, Dan Wang, Yuqing Guo, Steven Gregory, Leonardo Medico, Qiang Hu, Li Yan, Kunle Odunsi, Shashikant Lele, Song Liu

Yuqing Guo should have been included in the author byline as the fourth author.

The correct affiliations for this author are 1: Department of Cancer Prevention and Controls, Roswell Park Cancer Institute, Buffalo, New York, United States of America

The correct affiliations for this author are: Performed the experiemnts

The correct citation is: Zhao H, Shen J, Wang D, Guo Y, Gregory S, et al. (2012) Associations between Gene Expression Variations and Ovarian Cancer Risk Alleles Identified from Genome Wide Association Studies. PLoS ONE 7(11): e47962. doi:10.1371/journal.pone.0047962 

