# Supplementary figures and images for: Associations between Gene Expression Variations and Ovarian Cancer Risk Alleles Identified from Genome Wide Association Studies
Source: PLoS One. 2012 Nov 2;7(11):e47962. doi: 10.1371/journal.pone.0047962 (PMC3487879; doi:10.1371/journal.pone.0047962)

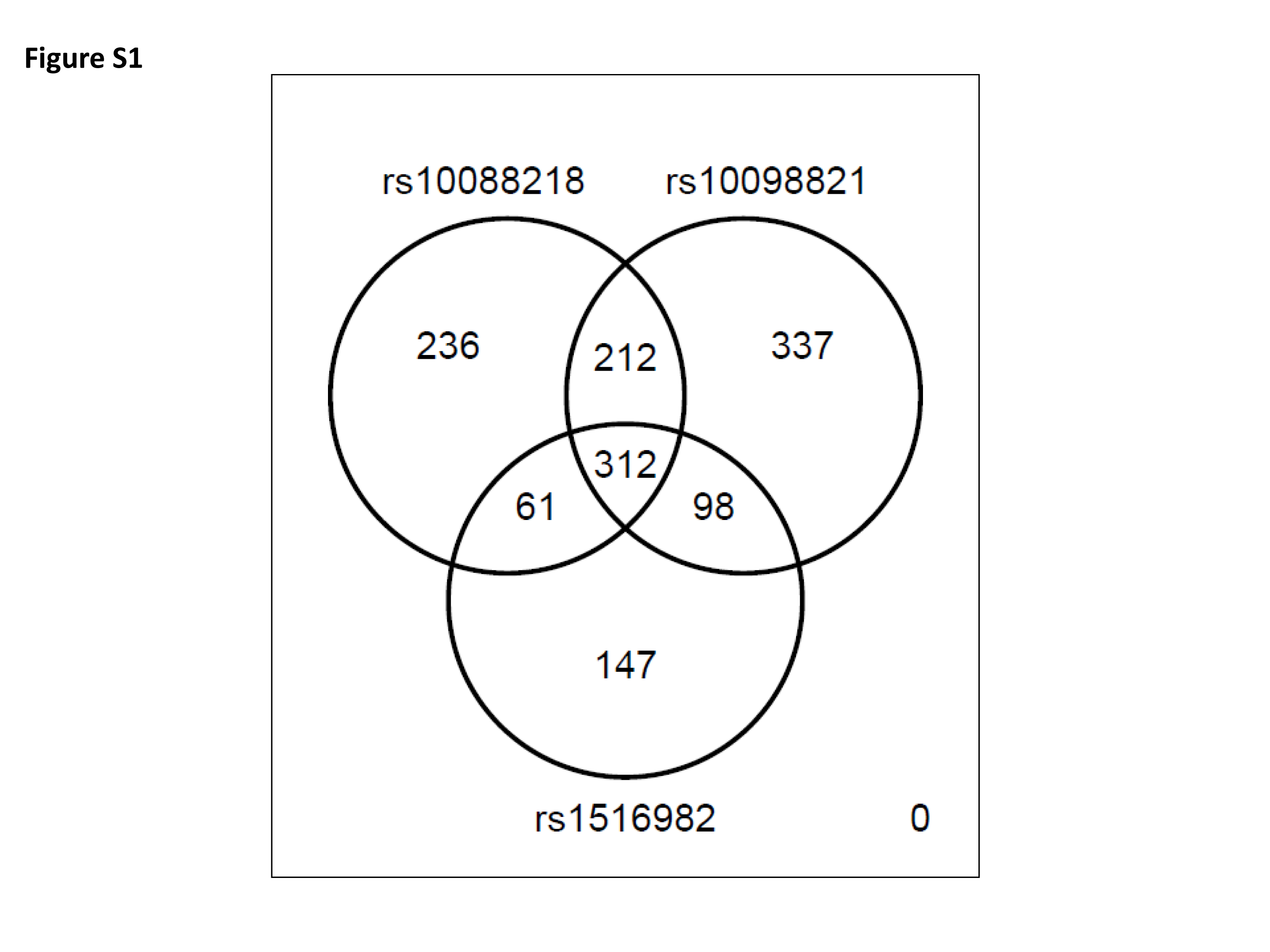

Supplement: Figure S1 — Venn diagram showing the overlaps of mRNA genes significantly (permutated P<0.05) associated with the three GWAS discovered SNPs from 8q24 locus. (TIF) [file pone.0047962.s001.tif]

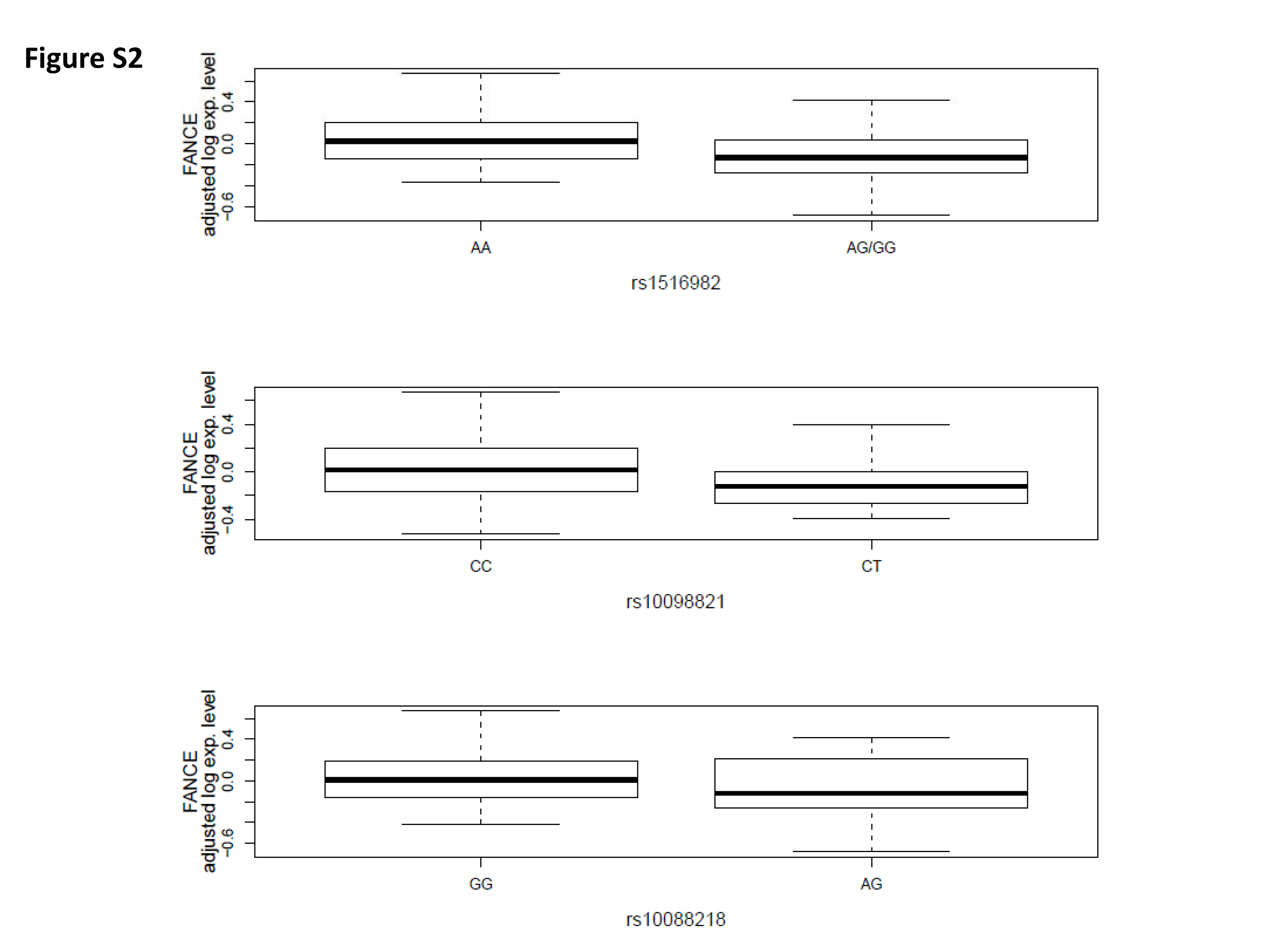

Supplement: Figure S2 — Significant associations between the genotypes of the three variants on 8q24 locus and FANCE expression phenotypes. The boxplot shows the relationship between log2 residuals of FANCE expression levels (adjusted for age and case-control status) and genotype of the rs1516982 (top, permutated P = 8.0×10^−4^, adjusted r2 = 10.3%), rs10098821 (middle, permutated P = 0.0037, adjusted r2 = 7.0%) and rs10088218 (bottom, permutated P = 0.0312, adjusted r2 = 3.4%). (TIF) [file pone.0047962.s002.tif]

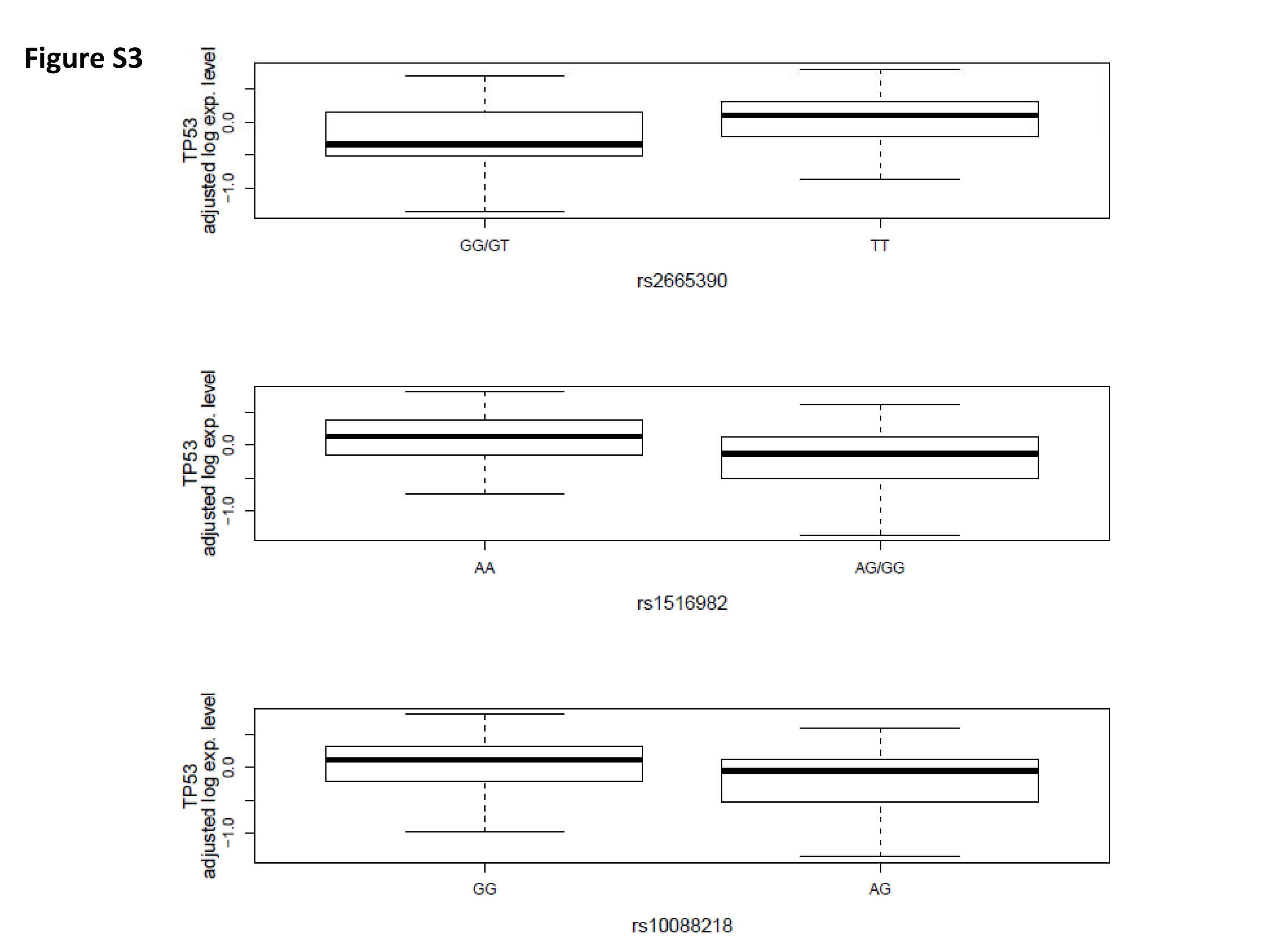

Supplement: Figure S3 — Significant associations between the variants genotypes and p53 expression phenotypes. The boxplot shows the relationship between log2 residuals of p53 expression levels (adjusted for age and case-control status) and genotype of the rs2665390 (top, permutated P = 0.0181, adjusted r2 = 3.6%), rs1516982 (middle, permutated P = 0.0279, adjusted r2 = 3.5%) and rs10088218 (bottom, permutated P = 0.0494, adjusted r2 = 2.5%). (TIF) [file pone.0047962.s003.tif]
